# Fast Reservoir Characterization with AI-Based Lithology Prediction Using Drill Cuttings Images and Noisy Labels

**DOI:** 10.3390/jimaging9070126

**Published:** 2023-06-21

**Authors:** Ekaterina Tolstaya, Anuar Shakirov, Mokhles Mezghani, Sergey Safonov

**Affiliations:** 1 Aramco Innovations LLC, 117127 Moscow, Russia; anuar.shakirov@aramcoinnovations.com (A.S.); sergey.safonov@aramcoinnovations.com (S.S.); 2 EXPEC Advanced Research Center Saudi Aramco, Dhahran 34466, Saudi Arabia

**Keywords:** drill cuttings, noisy labels, lithology prediction, machine learning

## Abstract

In this paper, we considered one of the problems that arise during drilling automation, namely the automation of lithology identification from drill cuttings images. Usually, this work is performed by experienced geologists, but this is a tedious and subjective process. Drill cuttings are the cheapest source of rock formation samples; therefore, reliable lithology prediction can greatly reduce the cost of analysis during drilling. To predict the lithology content from images of cuttings samples, we used a convolutional neural network (CNN). For training a model with an acceptable generalization ability, we applied dataset-cleaning techniques, which help to reveal bad samples, as well as samples with uncertain labels. It was shown that the model trained on a cleaned dataset performs better in terms of accuracy. Data cleaning was performed using a cross-validation technique, as well as a clustering analysis of embeddings, where it is possible to identify clusters with distinctive visual characteristics and clusters where visually similar samples of rocks are attributed to different lithologies during the labeling process.

## 1. Introduction

Lithology data are essential for many applied problems of petroleum engineering, including reservoir characterization, inter-well correlation, geological model construction, basin and petroleum system modeling, etc. The analysis of rock sample recovering during borehole drilling can be considered as one of the primary and direct sources of information about rock lithology. These samples are known as rock cuttings. The initial data on the lithological content of rock cuttings are obtained on-site by geologists that have relevant expertise for conducting cuttings description. More precise data on rock lithology are obtained by means of laboratory equipment (such as X-ray diffraction, X-ray fluorescence, near-infrared spectroscopy, etc.) after the drilling is completed and representative sample collection is delivered for detailed analysis in the laboratory or data, obtained during drilling [1,2,3,4].

Due to economic reasons, a common practice is to conduct detailed laboratory investigations of core samples that were recovered from the ‘sweet-spots’ that represent hydrocarbon-bearing intervals of the well. Thus, precise data on rock lithology are often constrained to some specific depth intervals. Moreover, some results of laboratory investigations may not be representative in case of a high heterogeneity of the drilled formations.

At the same time, drill cuttings offer a rich source of data that encompasses a broader stratigraphic range compared to full-sized cores. Analyzing them in near-real-time could enable cost-effective and fast reservoir characterization. Therefore, the development of an automated pipeline for an on-site description of drill cuttings lithology is a high-priority task for many petroleum companies. 

In this work, we researched the possibility of detecting lithology using just white light images, which are the cheapest data to obtain during drilling. The commonly used pipeline to infer data on rock lithology from cuttings is the following. During the drilling of a wellbore, a rotating bit breaks the rocks downhole, and drilling mud carries the broken fragments to the surface. At the surface, these fragments move to a shaker, which separates the drilling mud from the rocks for recirculation. Cuttings are cleaned from the drilling mud and a field geologist conducts a manual visual description of the recovered samples based on their expertise. 

The manual description of rock cuttings very often has the following problems: (1) biased descriptions due to the subjective nature of the results, (2) a time-consuming process, (3) human errors due to mud contamination of cuttings. Moreover, some types of rocks can be very close in terms of visual appearance, which complicates the process of visual description. One of the possible solutions for these problems is the implementation of an automated workflow for detecting rock lithology from rock cuttings.

Within our research, we developed and proposed a new approach for estimating the lithological composition of drill cuttings from digital photographs. The proposed method relies on the application of computer vision techniques and is data-driven. The basis for the approach development was a dataset comprising high-resolution images and results of drill cuttings description conducted by field geologists from more than 20 industrial wells. The dataset includes cuttings images for nine lithotypes, including clastic and carbonate rocks. We developed a convolutional deep learning model that predicts the likelihood of a sample belonging to a specific rock class. To understand the generalization ability of the developed model, we tested our approach on an unseen well.

Much effort has been invested in the cuttings images analysis, with some valuable research contributions that we will briefly outline. In [5], the authors suggested the application of several classification approaches to predict rock lithology from drill cuttings. The initial approach was to use a pre-trained AlexNet combined with a support vector machine classification of learned features. However, the validation results were below expectations. Thus, the authors tried to use a Bayesian-optimized network and network ensemble, which performed worse than a single network. They utilized a dataset from the 2019 EAGE hackathon, which is now unavailable. With a validation set of 34 images, they achieved an 80%+ accuracy but noted that some labels in the set might be erroneous. This highlights the challenge of accurately classifying drill cuttings containing rocks with different lithologies, as they can appear very similar and are prone to misclassification.

In [6], the authors focused on a specific case of drilling cuttings produced by polycrystalline diamond compact (PDC) bits, which yield small-sized pieces. The paper outlines a segmentation method for determining the quantity of oil-bearing cuttings in an image using a special type of fluorescent light. The segmentation was carried out using the watershed algorithm, and lithology was identified by comparing image features with known features from a feature library. However, this algorithm cannot provide the lithological composition of cuttings extracted from a well during drilling. Instead, it suggests comparing feature vectors with those in the available feature library of image areas. In [7], the authors proposed using data on measurements of elemental rock composition alongside images, but such data require the lab processing of samples.

An approach based purely on image classification was proposed in [8]. In this work, the authors proposed using a CNN with an Inception-3 encoder and distinguishing 10–15 lithology classes. They trained the model on images where the dominant lithology is available. Patches of 512 by 512 pixels were fed into the network during training, and the final prediction was obtained by averaging patches results on unseen images. 

The lithology identification of mixtures of different lithotypes was considered in [9]. To find the amount of each lithotype on the image, the authors used a segmentation network. Firstly, they trained it using single lithotype images and a constant segmentation mask and, after that, applied the trained network to the images of mixtures and computed the proportion of each lithotype on the photo. The authors noted the main challenge in the lithology detection of drill cuttings, namely very high intra-class variations and low inter-class variations. To overcome this difficulty, the authors split the classes into several subclasses based on the visual appearance of cuttings—grain size, color, etc.—resulting in a total class number of 72 instead of 3 initial rock types: carbonate, sandstone, and shale. The authors did not mention whether subclass annotation was performed manually or automatically. The authors mentioned the work of [10], where it was shown that it is possible to learn more universal representations using a sub-classes approach. The authors applied K-means clustering to image embeddings and used cluster labels as additional features to initial class labels for training a new model.

The work in [11] continued the idea of learning more universal representations by applying clustering to embeddings. The authors used different numbers of clusters, from 500 to 15,000 (having approximately 1000 classes in the initial dataset labeling). It was proposed to add the cluster labels as additional labels to train the new model on this dataset. 

In this work, we tried to improve drill cuttings classification using dataset-cleaning techniques, clustering for identifying possibly erroneous classifications, and oversampling for a better separation of visually similar images.

## 2. Materials and Methods

Within this section, we (1) develop a workflow for lithology prediction from drill cuttings images, (2) describe a suggested approach to eliminate samples with errors in labels, (3) demonstrate the architecture of the applied neural network, and (4) describe the approach used to assess the prediction abilities of the neural network.

### 2.1. General Workflow for Lithology Prediction from Drill Cuttings’ Images

**Figure 1:**
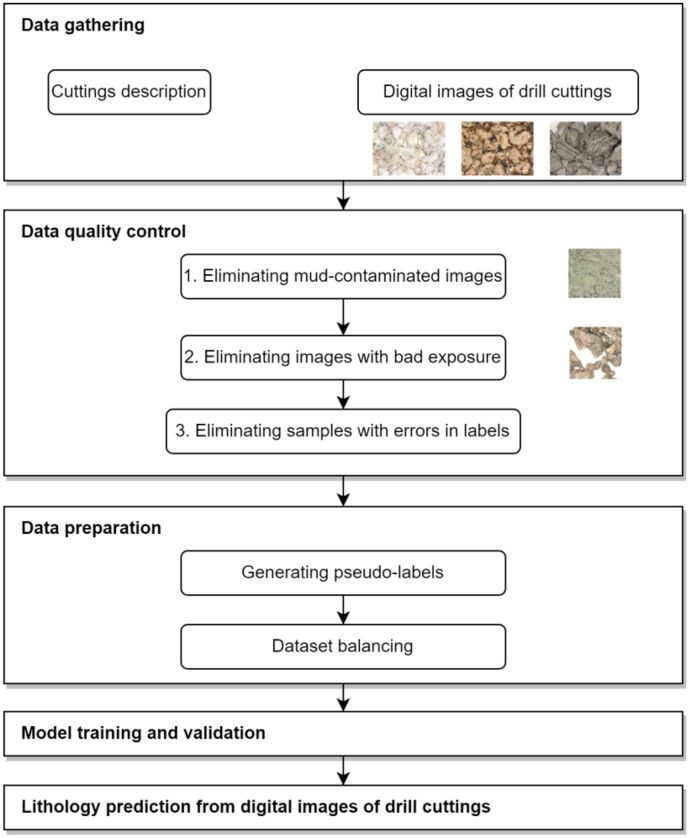
Workflow for predicting rock lithology from drill cuttings images by means of deep learning.

The developed workflow for predicting rock lithology from drill cuttings images is depicted in Figure 1 and includes the following principal steps:

1. Data gathering. The data include drill cuttings images and their labels. For labels, we imply lithology class provided by the field geologist. Cuttings should be taken under the same lightning conditions and fixed camera setup (shutter speed, aperture, sensitivity (gain), etc.). The samples of drill cuttings should be preliminary washed and dried to be informative with respect to their geological content.
2. Data quality control. Due to human errors, the data always contain some portion of samples with a poor quality of images or their labels. For poor-quality images, we assume images with mud contamination and images with bad exposure. To eliminate the images with mud contamination, a CNN with a binary output is used. Images with bad exposure can be detected by simple image processing techniques (e.g., assigning some threshold values for the luminosity). Errors during labeling is not a rare case due to (1) high variability of visual appearance within the same lithotype and (2) similarity between certain lithologies. The implemented approach for detecting samples with errors in sample labels is presented in Section 2.2.
3. Data preparation. This includes (1) generation of pseudo-labels and (2) data balancing. The generation of pseudo-labels is motivated by diversity of visual appearance within each lithotype. To generate pseudo-labels, we suggest utilizing clusterization of images based on their representations (embeddings). As in many other classification problems, data balancing is a crucial aspect to consider before model training. Depending on the geological profile type, some lithotypes are over-represented and others are represented in a limited quantity. The suggested balancing strategy is presented in Section 2.3.
4. Model training and validation. After the data are prepared, the model training and validation are conducted. To imitate the real case scenario, we subdivided the data into three subsets: (1) training subset, (2) validation subset, and (3) testing subset with unseen data from one well. The training and validation subsets are sampled randomly from the dataset (excluding testing subset) in the ratio of 80% and 20%, respectively. The model architecture is described in subsection “CNN model architecture”.
5. Lithology prediction from digital images of drill cuttings. Once the model is trained, it can be utilized for predicting rock lithology from digital images of drill cuttings. The results of predicting data for test well are described within the Section 3.

### 2.2. Detecting Samples with Wrong Labels

To identify the samples with errors in labeling, we relied on the following assumption. The dataset contains clusters with one dominant lithotype, and a minor portion of the data within each cluster are presented by samples with wrong labels. Thus, we conducted the quality control procedure in the following way:

1. Obtaining image embeddings. During our research, we used image embeddings in the form of fully connected hidden layer of the CNN.
2. Clustering image embeddings by means of Gaussian mixture algorithm (see, e.g., [12] by Ouyang et al.). To understand the optimal number of number of mixtures, we assessed the silhouette score [13], taking the maximum value of it.
3. Analyzing and processing the data within the detected clusters. During this step, we analyzed the frequency of the lithotype within the cluster. The samples with a low frequency were eliminated from the dataset.

Additionally, the samples should be sanitized for mud contamination and bad exposition during photographing of the samples. The mud-contaminated images can be identified by application of trained CNN that has a binary output (0 for clean sample and 1 for contaminated sample). The bad exposition of images can be determined by simple image processing techniques, e.g., we can calculate the percentage of images that have high values in RGB channels.

### 2.3. Data Balancing

**Figure 2:**
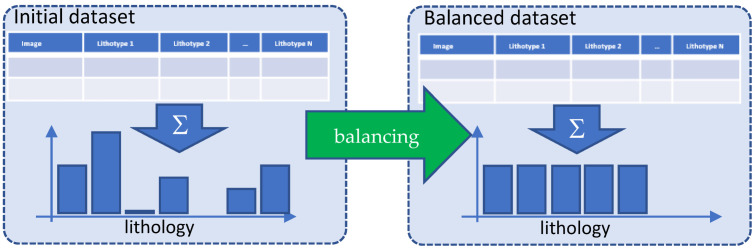
Dataset balancing.

The data balancing process schema is presented in Figure 2 and consisted of several principal stages (see [14]):

1. Selecting overrepresented lithotypes and removing from consideration a set of images with such lithotypes.
2. Finding unrepresented lithotypes (or present in negligibly small amount) and removing them from the dataset.
3. Determining the number to repeat (one or another sample) in the set so that, as a result, the total number of samples within each lithotype is approximately the same. We denote the number of repetitions by a vector **x**, where the length of **x** is equal to the number of remaining samples, and the **x** components are non-negative integers equal to the number of repetitions of a particular sample. From the label vector of remaining samples, we form a matrix **M**, where the columns are lithology vectors. The vector **x** can be found by minimizing the difference between quantities of different lithotypes:

$$\|Mx-{\langle Mx\rangle \|}_{L2}\;\overset{x}{\to }\min$$

It is worth noting that, when dealing with an imbalanced dataset, incorporating class weights during the training process can be useful. However, this may lead to a situation where an under-represented class rarely appears in the training batch for the network, and thus the network infrequently ‘encounters’ such a class during training. By repeating images of an under-represented class, it will appear in the data batches with the same frequency as the others, and additional image augmentation will aid in diversifying the visual representation of this class.

### 2.4. CNN Model Architecture

**Figure 3:**
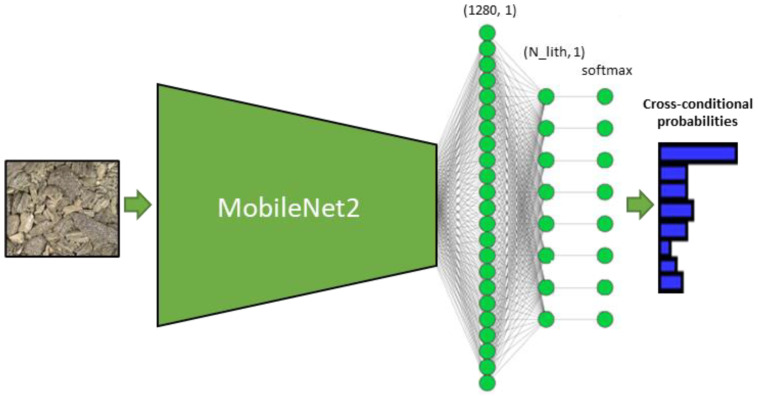
Deep learning model architecture. N_lith stands for total number of lithotypes to predict.

After testing distinct architectures of neural networks and analyzing the models’ performance and the resources required for model training, we selected the following architecture of the model (see Figure 3).

We selected MobileNet2 encoder, since it reportedly has good performance. Following [15], where the authors reported that “higher-capacity models perform better on the subset of incorrectly labeled test data in terms of their accuracy on the original labels”, we chose the architecture to be a compromise between size and performance, since our labels presumably contain errors, which are quite difficult to notice.

### 2.5. Assessment of Prediction Results

From comparison of true and predicted values of rock lithology, we calculated *accuracy* (Equation (1)), *recall* (Equation (2)), *precision* (Equation (3)), and *F*1 score (Equation (4)) for each lithotype separately. Additionally, we assessed macro and weighted average values for precision, recall, and *F*1 score.

(1) $$Accuracy=\frac{TP}{TP+FP+TN+FN}$$

(2) $$Recall=\frac{TP}{TP+FN}$$

(3) $$Precision=\frac{TP}{TP+FP}$$

(4) $$F1=2\cdot \;\frac{Precision\;\cdot Recall}{Precision+Recall}$$

where *TP* is true positive predicted values, *FP* is false positive predicted values, *TN* is true negative predicted values, and *FN* is false negative predicted values.

### 2.6. Dataset Overview

**Figure 4:**
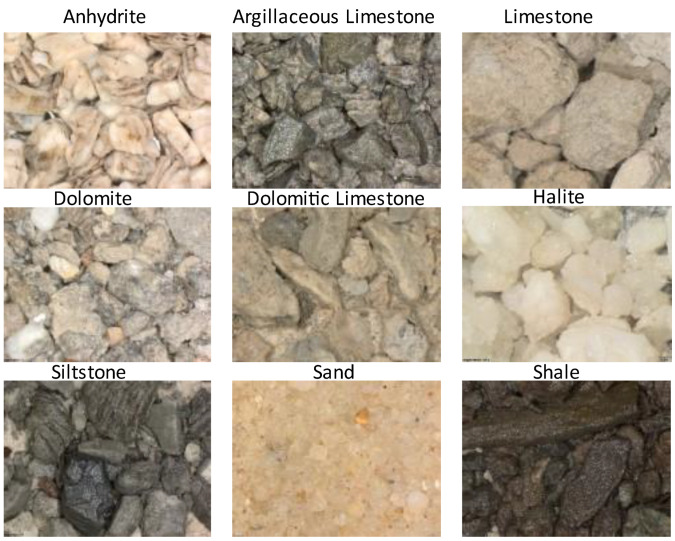
Examples of drill cutting images for different lithotypes.

Within our research, we gathered 16,687 cuttings images from 28 industrial wells in 22 distinct hydrocarbon fields that were captured under white light with a fixed camera set-up. The dataset contains the following lithotypes: (1) anhydrite (ANHY), argillaceous limestone (ARGLS), calcareous limestone (CALLS), clay, dolomite (DOL), dolomitic limestone (DOLLS), halite (HAL), limestone (LS), marl, sandstone (SAND), shale (SH), and siltstone (SST). Figure 4 illustrates several examples of drill cuttings images and a microscope used for making images.

Although external lighting conditions may slightly change during the photography process, artificial light sources were employed to minimize these variations. The camera parameters were manually set and remained constant, ensuring that shutter speed, aperture, and color representation in the images were not significantly altered. The depth from which each sample of drill cutting was retrieved was encoded in the file name. 

**Figure 5:**
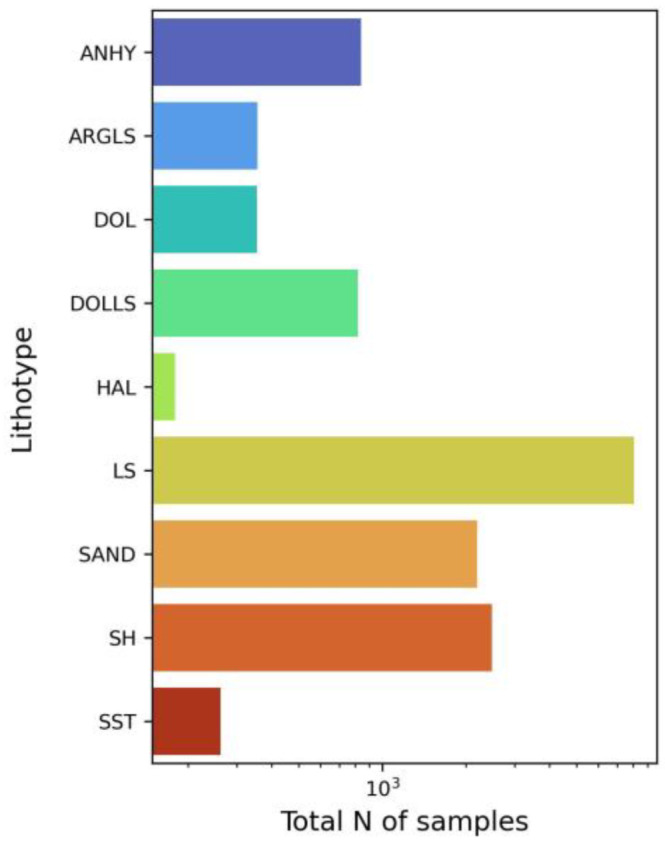
Bar plot representing total number of samples per lithotype within the dataset.

The gathered dataset exhibits a high degree of imbalance with respect to the total number of samples within distinct lithotypes (Figure 5). That imbalance is common for the study region. As can be seen from Figure 5, the dominant lithology is limestone, whereas the total number of samples for marl, clay, calcareous limestone, halite, siltstone, and dolomite is less than 1000. 

**Figure 6:**
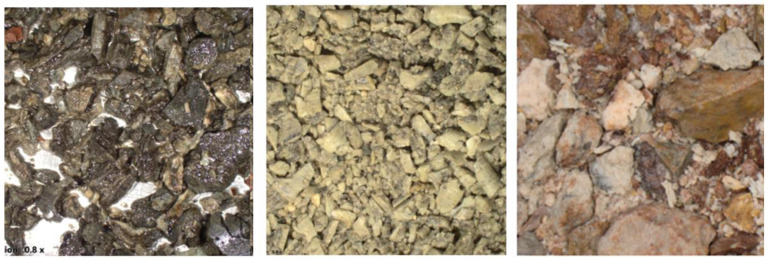
Example of distinct visual appearance for siltstone coming from different depth intervals and formations.

As was mentioned, the data come from distinct wells drilled through 101 geological formations occurring at different depth intervals. Due to different sedimentation environment within specific formations, there is a diversity of visual appearance of the same lithotypes coming from distinct formations. Figure 6 shows an example of siltstone samples coming from distinct depth intervals and formations. This problem conditions the necessity to generate pseudo-labels within each class to account for the domain specifics.

## 3. Results and Discussion

To evaluate the effectiveness of the proposed workflow, we conducted a set of experiments with different approaches to data preparation and model training. For testing the obtained models, we used data from an unseen well that was not used during training or validation. These experiments were:

- Baseline model. The dataset is taken as is and the model output contains only original lithology labels.
- Model training on sanitized dataset (removing mud-contaminated images, images with bad exposition, and samples with errors detected my means of the method described in Section 3.2).
- Model training on sanitized dataset and generated sub-labels by means of data clustering.

During model training, we used the adaptive moment estimation algorithm with a fixed learning rate of 0.0001. During model training, we set the batch size (number of samples processed before the model is updated) to 50 samples.

### 3.1. Training Baseline Model

**Figure 7:**
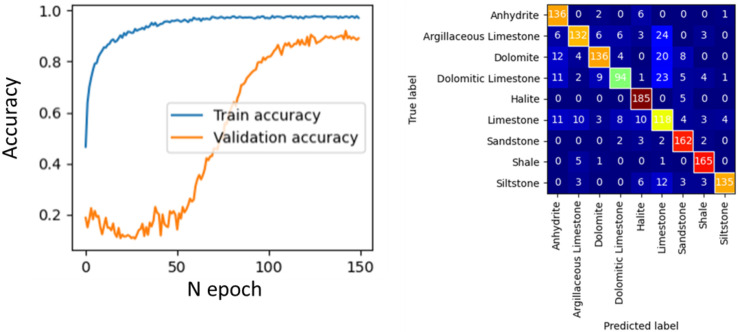
Accuracy curves for classifier training on 100% single lithotypes images (**left panel**); confusion matrix (**right panel**).

During the first experiment, the model for lithology prediction from cuttings images was trained without any data quality control and preprocessing. For training and validation, we took all images and randomly split them into train and validation subsets from the same wells. We applied standard augmentation techniques on the images, including rotation, shift, and skew with little color modification. The training history is depicted in the left panel of Figure 7, whereas the confusion matrix for the validation subset is presented in the right panel.

The obtained results allow us to conclude that the model converged well, with increasing accuracy curves (Figure 7). The prediction accuracy for validation images in each well ranged between 70% and 95%. As depicted in Figure 7, the model demonstrates strong generalization capabilities when both training and validation images are from the same set of wells. 

### 3.2. Results of Dataset Quality Control

To detect images with bad exposure, we specified threshold values for the percentage of pixels that are ‘too white’ or ‘too black’. For ‘too white’ images, we calculated the percentage of pixels that have minimum values along each RGB channel greater than 254. The threshold value of the percentage was set to 3.5% for considering the image as overexposed. For ‘too black’ images, we calculated the percentage of pixels that have maximum values along each RGB channel less than 10. The threshold value of the percentage was set to 10% for considering the image as underexposed. These threshold values were selected iteratively controlling the visual appearance of the images.

**Figure 8:**
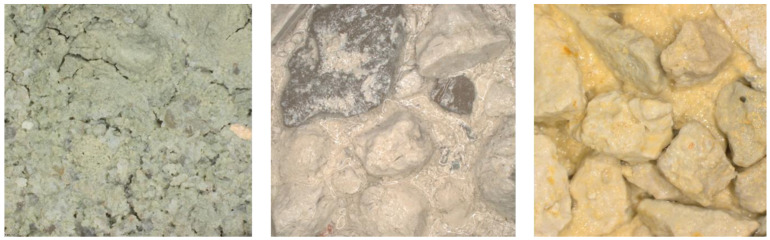
Examples of bad images with mud contamination.

To identify images of rocks covered with mud (examples illustrated in Figure 8), we first manually selected a subset of such contaminated images and trained a binary classifier that predicted mud contamination. After that, this model was applied to the whole dataset and revealed images with contamination. The total percentage of images detected as contaminated was 5% from the overall dataset, including the test well.

**Figure 9:**
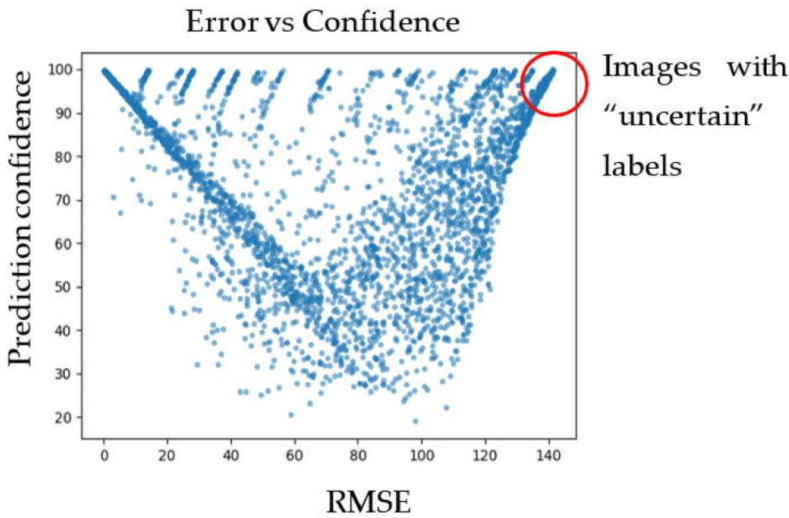
Plot of RMSE error versus prediction confidence for one of the cross-validation runs.

After that, we detected samples with problems with labeling. First, we applied a cross-validation technique: we divided all data into ten folds and performed training ten times, each time using one of the folds for model evaluation and the rest of the images for training. The uncertainly labeled images were those with a maximal error (when the model predicted a 100% single-lithotype image with the wrong lithotype) and highest predicted confidence. Figure 9 shows the plot of root mean square error (RMSE) versus prediction confidence for one of the runs. (Note that, in case a 100% single lithotype was misclassified, $\mathrm{RMSE}=100\sqrt{2}$.) The uncertain images for all runs of cross-validation were marked as having uncertain labels.

**Figure 10:**
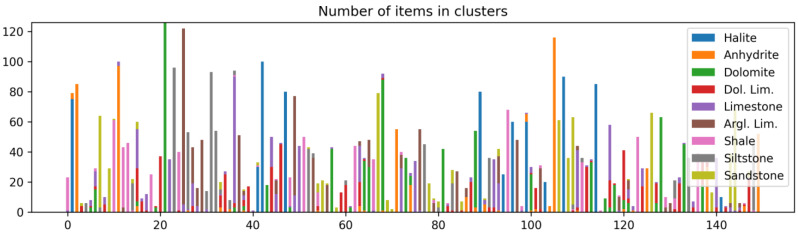
Distribution of lithotypes in clusters.

The model trained on sanitized data, after cross-validation, was used for image evaluation. To estimate how reliable the classification result can be, we applied clustering of the vectors embeddings of train images produced by this model. We applied the K-means clustering algorithm, where the number of clusters was estimated using the silhouette score after several clustering runs. The following Figure 10 illustrates the distribution of lithotypes in different clusters, where the number of clusters is equal to 150. The bar plot in Figure 10 shows the counts of samples in each cluster, and the color of the bars is attributed to different lithotypes. From this bar plot, we can see that each cluster can contain different lithotypes and a different count of samples of the lithotypes. 

**Figure 11:**
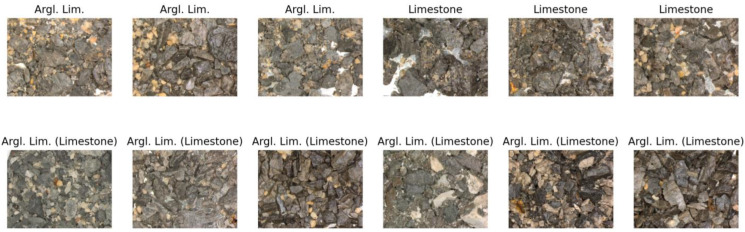
Cluster with 72 samples of argillaceous limestone and 17 samples of limestone (**top row**, from train set; **bottom row**, from test set; true label in brackets). Here, visually similar images have different labels; therefore, labeling is not reliable.

**Figure 12:**
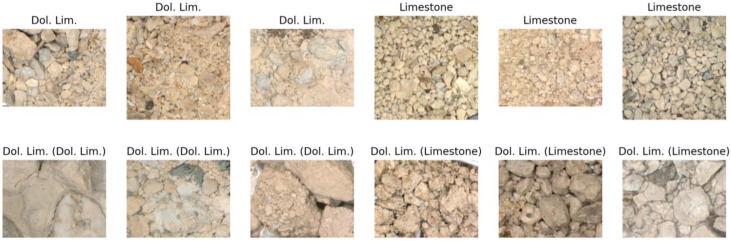
Cluster with 86 samples of dolomitic limestone and 83 samples of limestone (**top row**, from train set; **bottom row**, from test set; true label in brackets). Here, visually similar images have different labels; therefore, labeling is not reliable.

**Figure 13:**
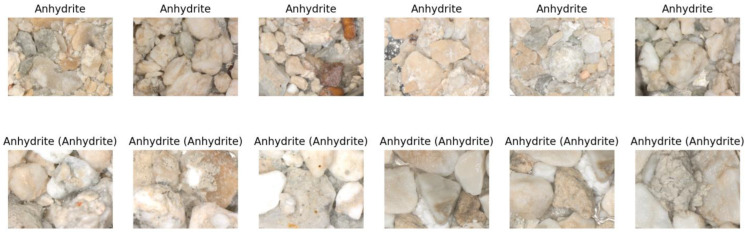
Cluster where the amount of most represented lithotype is larger (in our experiment, 10 times larger) than second-most represented lithotype, providing good classification results (**top row**, from train set; **bottom row**, from test set; true label in brackets).

The clusters contain varying quantities of lithotypes, while images within each cluster exhibit visual similarities. Usually, visual similarity between two images can be estimated as the Euclidean distance between vectors embeddings. Consequently, we can assess the reliability of labels for each cluster. To accomplish this, we identified the lithotype corresponding to the maximum and second maximum number of images within a specific cluster. If the maximum number is substantially larger than the second maximum, we have a relatively uniform cluster, indicating that visually similar images are labeled with the same lithotype. To attribute a cluster to a set of ‘good’ or ‘bad’ clusters, we used a threshold of 0.1, meaning that if the maximum is 10 times greater than the second maximum, we count the cluster as ‘good’; otherwise, it is ’bad’ (non-reliable). The following figures (Figure 11, Figure 12 and Figure 13) illustrate several clusters: on the top row, images from the train set are given; on the bottom row, images from the test set with the predicted lithotype using the dominant lithotype of the particular cluster, where the true label is in brackets, are given.

The prediction accuracy that we obtained using the clustering method is presented in Table 1 below. 

We can see that the prediction accuracy significantly drops in clusters where several lithotypes are present. In this case, we can ask for a re-labeling of these images or drop them from the training set.

### 3.3. Generation of Sublabels for the Dataset

**Figure 14:**
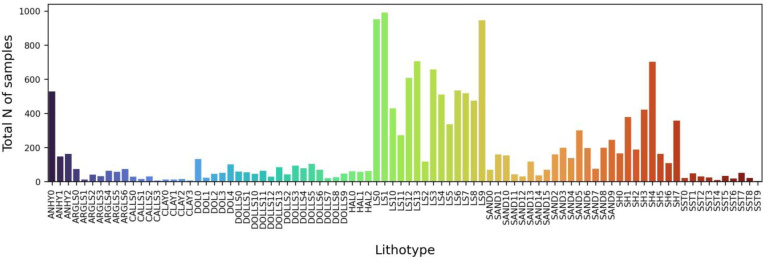
Bar plot representing total number of samples per sub-cluster of lithotypes.

Another approach that we applied is based on creating label sub-classes. To account for diversity within each lithotype, we relabeled our samples based on clustering in the following way. Firstly, we obtained embeddings for each sample by means of the baseline model. The shape of embeddings was 1280 × 1. Secondly, we conducted a dimensionality reduction of these embeddings by means of principal component analysis implemented within the scikit-learn library [16] to the shape 100 × 1. In the third step, we clustered the obtained embeddings within each lithotype separately using the Gaussian mixture algorithm. To detect the optimal number of clusters, we specified the range constraining the maximum of possible clusters to the total number of geological formations where the lithotype occurred. The optimal value of clusters was determined by the maximum value of the above-mentioned silhouette score. The results of data clustering are summarized within Table 2. The total number of samples within each cluster is depicted in Figure 14.

### 3.4. Data Balancing and Model Training

**Figure 15:**
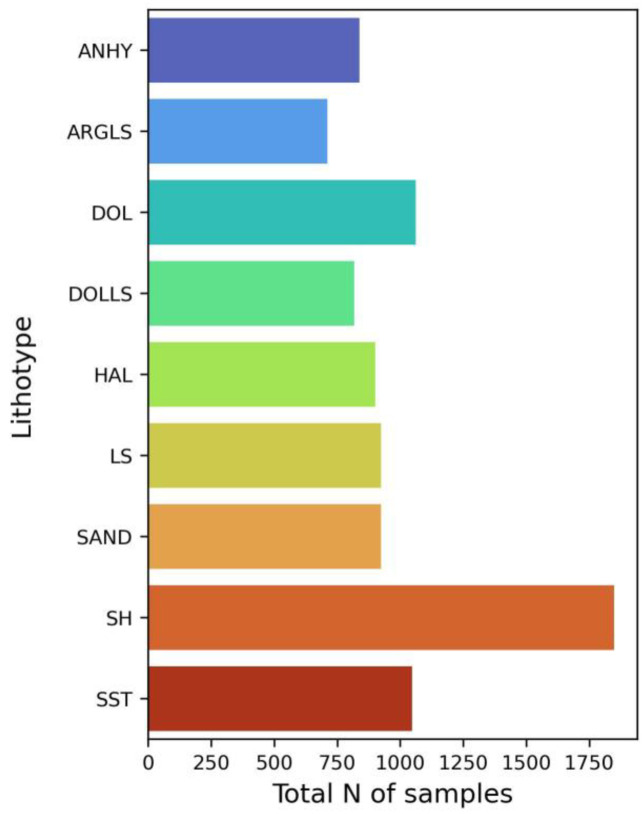
Results of data balancing for original dataset.

**Figure 16:**
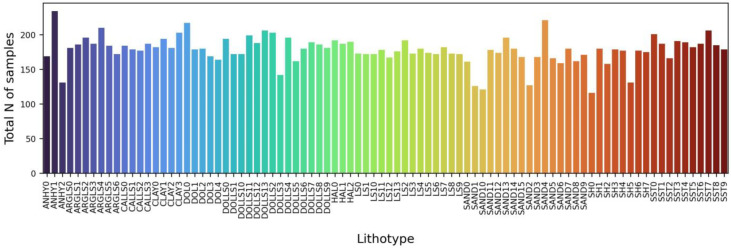
Results of data balancing for dataset with sublabels. Numbers after lithology short names indicate the relevance to the clusters.

After the data quality control and generation of sublabels, data balancing was carried out following the general workflow (see Figure 2). The applied algorithm for data balancing is described within Section 2.3. Figure 15 illustrates the results of data balancing for the dataset without the additional generation of sublabels. Figure 16 depicts the results of data balancing for the dataset with additionally generated sublabels.

**Figure 17:**
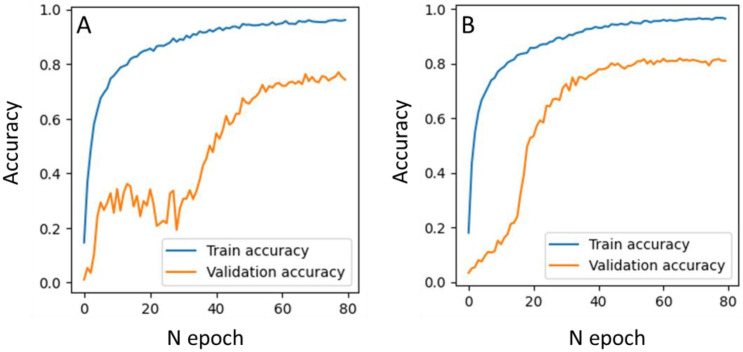
Accuracy curves for classifier training on the dataset without (**A**) and with (**B**) additional sublabels after data balancing.

Model training history for data without and with the generation of sublabels for lithotypes is presented in Figure 17. As can be seen from Figure 17, the generation of sublabels resulted in a faster convergence of the prediction accuracy for the validation subset.

### 3.5. Predicting Rock Lithology for Samples from Unseen Well

The three trained models (the baseline model, the model trained on the sanitized dataset, and the model trained on the sanitized dataset with the generation of sublabels within lithotypes) were applied to predict rock lithology from cuttings images of the unseen well. From the comparison of the true and predicted classes of lithotypes, we assessed the performance of models (Table 3).

From the obtained results, we can conclude that, when the baseline model is applied to images from a different well not used in training and validation, the model’s performance deteriorates significantly (28% accuracy). In this case, the lithology estimation is rather coarse, with some lithotypes predicted more inaccurately than others. This could be attributed to varying conditions for sample preparation, rock grinding, and drilling fluid composition, and a significant diversity in the training set. From these results, we can see that the whole dataset contains a lot of uncertainties, which limit the models’ performance.

At the same time, the conducted quality control of the dataset resulted in a significant enhancement of the model quality (the accuracy increased to 51). This evidences that quality control is one of the key aspects enhancing the generalization ability of the model. 

The generation of sublabels allowed for accounting for diversity within each lithotypes and resulted in an additional increase in prediction metrics (to a 60% accuracy). The obtained results correlate with research findings presented in [10,11].

## 4. Conclusions

A new approach for predicting rock lithology from cuttings images was developed and tested on experimental data from 28 industrial wells. The developed approach relies on the application of a convolutional neural network. The utilized CNN architecture represents an adaptation of the MobileNet2 for the problem. A special data preparation workflow was implemented within the experiment to account for (1) the diversity of visual appearance within each lithological class and (2) errors in image labels. The diversity of the visual appearance was accounted for through clustering each lithotype separately and generating new sub-classes based on the clustering results. The errors in image labeling were detected based on the results of clustering the whole dataset and analyzing the classes distribution within the obtained clusters. Within the case study, we trained three models: (1) a baseline model, (2) a model trained on a sanitized dataset, and (3) a model trained on a dataset with new sublabels generated by means of clustering. The performance of the models was assessed on data from an unseen well. The obtained results reveal a significant increase in prediction metrics due to the data preparation step. This evidences that the data preparation step plays one of the key roles during the enhancement of the generalization ability of the model for predicting cuttings lithology. The model trained on generated sublabels via clustering also provided a prominent increase in prediction metrics for the unseen well. The obtained results show that, in the best case, the F1 score on the unseen well can reach 66%, which is an acceptable level of prediction uncertainty. The implementation of the developed workflow can sufficiently reduce the time spent by geologists on cuttings description and can eliminate human errors. The next step for the research direction could be testing and enhancing the proposed solution for samples with mixed lithology. This problem can be classified as regression-type and will obviously require additional efforts. 

## Figures and Tables

**Table 1:** Lithology prediction accuracy using clustering.

	Train	Test	Samples Number
‘Good’ clusters	98%	51%	5451
‘Bad’ clusters	70%	28%	3476

**Table 2:** Results of data clustering within each lithotype.

Lithotype	N of Formations	Optimal N of Clusters
Anhydrite	5	3
Argillaceous limestone	10	7
Calcareous limestone	6	4
Clay	5	4
Dolomite	9	5
Dolomitic limestone	18	14
Halite	6	3
Limestone	17	14
Marl	1	1
Sandstone	20	16
Shale	11	8
Siltstone	15	10

**Table 3:** Results of predicting rock lithology for cuttings images from unseen well.

Lithotype	*Precision*	*Recall*	*F*1		*Accuracy*		
	Macro Avr	Weighted Avr	Macro Avr	Weighted Avr	Macro Avr	Weighted Avr	
Baseline model	0.26	0.78	0.29	0.46	0.23	0.56	0.28
Sanitized data model	0.32	0.71	0.40	0.5	0.30	0.61	0.42
Sub-labels model	0.36	0.77	0.45	0.6	0.34	0.66	0.60

## Data Availability

The data are unavailable due to privacy restrictions.
